# Prevalence and predictors of liver disease in HIV-infected children and adolescents

**DOI:** 10.1038/s41598-017-11489-2

**Published:** 2017-09-26

**Authors:** Maria Pokorska-Śpiewak, Aleksandra Stańska-Perka, Jolanta Popielska, Agnieszka Ołdakowska, Urszula Coupland, Konrad Zawadka, Małgorzata Szczepańska-Putz, Magdalena Marczyńska

**Affiliations:** 1 Department of Children’s Infectious Diseases, Medical University of Warsaw, Warsaw, Poland; 2 Hospital of Infectious Diseases, Warsaw, Poland

## Abstract

Liver disease in HIV-infected patients may result from the infection itself, antiretroviral treatment or comorbidities. In this study, we analysed liver disease in 79 HIV-infected children and adolescents aged 14.0 ± 5.1 years. All the patients were receiving combination antiretroviral therapy (cART), with a mean duration of 11.5 ± 4.7 years. Six patients (8%) had detectable HIV viral load, and 8/79 (10%) of the participants were coinfected with hepatitis B or C virus (HCV, 6/8 or HBV, 2/8). Liver disease was defined as an elevation of any of the following parameters: alanine or aspartate aminotransferase (ALT and AST), total bilirubin, and gamma glutamyl transferase (GGTP). For the noninvasive evaluation of liver fibrosis, the AST-to-Platelet Ratio Index (APRI) and Fibrosis-4 (FIB-4) were calculated. Liver disease was diagnosed in 20/79 (25%) of the patients, including 13/71 (18%) of participants without coinfection and 7/8 (88%) with coinfection (p < 0.0001). All of the liver markers except bilirubin were significantly higher in the coinfected group. APRI scores indicated significant fibrosis in 5/8 (63%) of patients with coinfection. HBV or HCV coinfection and detectable HIV viral load were independently positively associated with APRI (p = 0.0001, and p = 0.0001) and FIB-4 (p = 0.001, and p = 0.002, respectively). In conclusion, liver disease in HIV-infected children and adolescents results mainly from HBV or HCV coinfection. Effective antiretroviral treatment is protective against hepatic abnormalities.

## Introduction

In the era of combination antiretroviral therapy (cART), the risk of Acquired Immunodeficiency Syndrome (AIDS)-associated morbidities and mortality has decreased significantly and has been replaced by illnesses and deaths resulting from non-AIDS causes^1–3^. Liver disease has emerged as the most common cause of death in HIV-infected adults in United States, Europe, and Australia, accounting for 14–18% of all deaths^1,2,4^. According to the limited available data in pediatric patients, hepatitis does not seem to be as common cause of death in HIV-infected children as in adults^3^. Liver disease in HIV-infected patients may result from the infection itself, antiretroviral drug toxicity, or comorbidities, including coinfection with hepatitis B and C viruses (HBV and HCV)^5^. HIV-infection is considered a cause of many hepatobiliary disorders, including elevated liver enzymes, hepatomegaly and liver steatosis^6^. The possible mechanisms for HIV-related liver injury include a direct interaction between HIV and multiple liver cell types and an influence of HIV glycoproteins on hepatic stellate cells resulting in the stimulation of collagen production^6–8^. Before the cART era, opportunistic infections and AIDS-related neoplasms were the most common causes of liver injury in HIV-infected patients^4,9,10^. After the broad implementation of cART, the spectrum of liver disease in HIV-infected patients has shifted to medication-related hepatotoxicity, concomitant infections with HCV and HBV, non-alcoholic fatty liver disease, and alcohol abuse^4,11,12^.

Liver biopsy is considered the gold standard for assessing the presence and degree of liver inflammation and fibrosis^13,14^. However, due to its invasive nature, several noninvasive methods, including two serum biomarkers, Aspartate transaminase to platelet ratio index (APRI), and Fibrosis-4 (FIB-4) have been proposed^15^. Both markers were validated for their ability to detect liver fibrosis in adult patients with viral hepatitis^15^. However, there is only limited experience with these markers in HIV-infected children^5,6,16,17^.

Currently, HIV-infected children survive into adulthood and face lifelong infection and treatment. Thus, liver disease may emerge as an important risk factor for morbidity and mortality in HIV-infected paediatric patients^17^. However, data on hepatic dysfunction in HIV-infected children are limited, and studies from European cohorts are lacking in this field^5,6,16,17^.

Thus, the aim of this study was to analyse the prevalence and predictors of liver disease in a regional cohort of HIV-infected children and adolescents receiving cART. In addition, the available noninvasive biomarkers of liver disease were determined.

## Methods

### Patients and laboratory evaluation

Our tertiary health care department takes care of over 70% of all HIV-infected children and adolescents up to 20 years of age in Poland. All children with confirmed HIV infection are treated with cART; we have no treatment-naïve patients. In this retrospective observational study we included all patients aged 2–20 years, who acquired HIV infection during childhood. Data were obtained during the five-year period, between 2012 and 2016. We recorded data from the last follow up in 2016, when available. For patients who were referred to the adult outpatient unit before 2016, data from their last visit were analysed. Patients with other well-established causes of liver disease, such as Wilson’s disease, alpha 1-antitrypsin deficiency, autoimmune hepatitis, or non-alcoholic fatty liver disease (NAFLD), were excluded from this study. Probable dates and modes of HIV infection were determined based on the available medical records. The putative age when the infection was acquired and the duration of the disease were calculated from the beginning of risk exposure. HIV infection was diagnosed according to the current Polish recommendations, concordant with the World Health Organization (WHO) guidelines, and confirmed using HIV RNA nucleic acid testing (Real Time HIV-1, Abbott)^18^. The limit of detection of HIV RNA assay was 40 copies/ml. In all patients, serological and molecular testing for hepatitis B virus (HBV) and hepatitis C virus coinfection was performed. Concomitant hepatitis B was diagnosed based on positive hepatitis B surface antigen testing, as confirmed by positive HBV DNA polymerase chain reaction (PCR), whereas hepatitis C diagnosis was made using anti-HCV testing and confirmed by nucleic acid testing - positive HCV RNA real-time PCR. Serological determinations were performed using commercially available ELISA kits (Vitros ECi, Ortho-Clinical Diagnostics, Johnson&Johnson), and for nucleic acid testing, Amplicor (Roche) and Cobas TaqMan (Roche) were used.

Each patient’s evaluation included medical history, physical examination with recording of patients’ weights and heights, standard haematologic and biochemical assays, CD4 count, and HIV RNA viral load. The following biochemical liver markers were determined using commercially available laboratory kits (Vitros 5600, Ortho-Clinical Diagnostics, Johnson & Johnson): alanine aminotransferase (ALT), aspartate aminotransferase (AST), bilirubin, and gamma glutamyltransferase (GGTP). Upper limits of normal (ULN) were assessed at 40 IU/L for ALT and AST, 22 µmol/L for bilirubin, and 73 IU/L for GGTP. Liver disease was defined as any elevation of any of the parameters above the ULN. BMI *z*-scores were calculated according using the WHO Child Growth Standards and Growth reference data with the WHO Anthropometric calculator AnthroPlus v.1.0.4.

### Evaluation of liver fibrosis

For the evaluation of liver fibrosis, noninvasive serum biomarker analysis was performed, which included calculating the AST to platelet ratio index (APRI) and Fibrosis-4 score (FIB-4) according to the published analytic recommendations^19,20^, using following equations (1, 2):

1 $${\rm{APRI}}=[{\rm{AST}}({\rm{IU}}/{\rm{L}})/{\rm{AST}}\,{\rm{ULN}}({\rm{IU}}/{\rm{L}})/{\rm{platelet}}\,{\rm{count}}({10}^{9}/{\rm{L}})]\times 100;$$

2 $$\mathrm{FIB}-4=[{\rm{age}}\,({\rm{years}})\times {\rm{AST}}({\rm{IU}}/{\rm{L}})]/[{\rm{platelet}}\,{\rm{count}}\,({10}^{9}/{\rm{L}})\times \sqrt{\mathrm{ALT}(\mathrm{IU}/{\rm{L}})}].$$

According to the previously published data, the following cut-offs for the biomarkers were considered: APRI >0.5 and FIB-4 >1.45, which suggested significant fibrosis, and APRI > 1.5, which suggested cirrhosis^20,21^.

### Statistical analysis

Continuous variables were tested for normal distribution using the Kolmogorov-Smirnov test and were expressed as the mean ± standard deviations (SD) or medians with interquartile ranges (IQR), as appropriate. Continuous data were compared using Student’s t-test or the Mann-Whitney test, whereas the categorical variables were analysed with either the chi-square test or Fisher’s exact test.

A linear regression analysis was conducted to identify the predictors of the liver disease, and Pearson correlation coefficients were obtained. Six separate multiple regression models were constructed for the following markers of the liver disease: ALT, AST, bilirubin, GGTP, APRI, and FIB-4. Candidate predictors (age, sex, BMI z-score, CD4 count, duration of cART, haemoglobin level, platelet count, total cholesterol, HBV or HCV coinfection, HIV viral load, and previous AIDS diagnosis) were entered into the model, regardless of the results of the univariate analysis. After entering all variables to the model, the variables that showed least significant associations were subsequently excluded until all variables remained significant (p < 0.05). The model fit for the multiple regression was assessed using the *R* ^2^ - coefficient of determination and the adjusted *R* ^2^ - coefficient of determination, adjusted for the number of independent variables in the model.

To investigate the influence of different antiretroviral regimens and drugs on liver function parameters, a logistic regression analysis was performed. Since the known coinfection with hepatotropic viruses could have influenced the chosen antiretroviral regimen, only patients without coinfection were included in this part of the analysis.

A two-sided p-value of <0.05 was considered to indicate significance. All statistical analyses were performed with the use of MedCalc Statistical Software ver. 17.2 (MedCalc, Mariakerke, Belgium).

### Ethical statement

The investigation was concordant with the principles outlined in the Declaration of Helsinki and its future amendments. The local Bioethics committee at the Medical University of Warsaw, Poland approved the project of this study. Written informed consent was collected from all of the patients and/or their parents/guardians for participation in the study.

## Results

### Patient characteristics

Seventy-nine patients (40 male and 39 female) aged 14.0 ± 5.1 years (the range 2–20 years), with a mean duration of cART of 11.5 ± 4.7 years, were enrolled in this study. Eight patients (10%) were coinfected with either HBV (2/8) or HCV (6/8). Most of the participants (96%) were infected vertically. All patients with HIV/HBV and HIV/HCV coinfection were simultaneously infected with both viruses. Seventy-six patients (96%) did not show immunosuppression (stage 1 according to the Centers for Disease Control and Prevention, CDC classification), and only 3/79 (4%) of the participants had moderate suppression (stage 2 by CDC, all three patients with detectable HIV viral load due to the poor adherence)^22^. The HIV viral load was undetectable or below 40 copies/mL in 73/79 (92%) of patients. In the remaining 6/79 (8%) of the participants, the HIV viral load was between 210 and 2056 copies/mL. In all these cases, detectable viremia was due to an adherence problem. A previous AIDS diagnosis was reported in 25/79 (32%) of cases. At the time of analysis, 38/79 (48%) of the patients were receiving a regimen containing two nucleoside reverse transcriptase inhibitors (NRTI) and a non-nucleoside reverse transcriptase inhibitor (NNRTI), (including 18/79, 23% receiving nevirapine), for 27/79 (34%) it was a second line therapy; 29/79 (37%) were receiving 2 NRTIs and a protease inhibitor (PI), (16/79, 20% second line), and the remaining 12/79 (15%) patients were receiving integrase inhibitor (INSTI)-based therapy (all of them second/third line). HBV-coinfected children received two NRTIs active against HBV. The most common causes of switching to second line were side effects of cART (no cases related to liver disfunction), regimen simplification to fixed dose combination, or lack of availability of specific drugs. Coinfected patients were significantly older compared to patients without coinfection (17.7 ± 2.5 vs. 13.6 ± 5.2 years) and had a longer cART duration (15.5 ± 3.5 vs. 11.0 ± 4.6 years). Baseline demographic and laboratory characteristics of the study group including patients with and without HBV or HCV coinfection are presented in Table 1.

**Table 1:** Baseline demographic and laboratory characteristics of the study group.

Characteristics		Total	HIV without HBV or HCV coinfection (Group I)	HIV with HBV or HCV coinfection (Group II)	P Group I vs. Group II
Number		79	71	8	
Sex	Male (%)/Female (%)	40 (51)/39 (49)	34 (48) / 37 (52)	6 (75)/2 (25)	0.26
Age (years)	Mean ± SD	14.0 ± 5.1	13.6 ± 5.2	17.7 ± 2.5	0.03
Duration of cART (years)	Mean ± SD	11.5 ± 4.7	11.0 ± 4.6	15.5 ± 3.5	0.008
BMI z-score	Mean ± SD	0.06 ± 1.0	0.003 ± 1.0	0.53 ± 1.1	0.16
Mode of infection	Vertical (%)	76 (96)	69 (97)	7 (88%)	0.27
HIV viral load	Number (%)				
	≥40 copies/mL	6 (8)	5 (7)	1 (13)	0.48
	<40 copies/mL	73 (92)	66 (93)	7 (87)	
CD4+ cell count	Median (IQR)	842 (643–1039)	883 (631–1052)	784 (670–859)	0.35
	%	50 (44–55)	49 (43–55)	52 (50–55)	0.13
CDC classification	Stage 1	76 (96)	68 (96)	8 (100)	1.0
	Stage 2	3 (4)	3 (4)	0 (0)	
History of AIDS	Number (%)	25 (32)	23 (32)	2 (25)	1.0
Laboratory findings	Hemoglobin (g/dL)	14.3 ± 1.3	14.3 ± 1.4	15.0 ± 0.8	0.13
Mean ± SD	Platelets (×10^9^/L)	265.8 ± 70.5	272.0 ± 68.6	211.8 ± 67.8	0.02
	Cholesterol (mg/dL)	178.0 ± 45.3	179.6 ± 39.2	167.0 ± 97.6	0.51
cART regimen type Number (%)	2 NRTI + NNRTI	38 (48)	32 (45)	6 (76)	0.14
	2 NRTI + PI	29 (37)	28 (39)	1 (12)	
	INSTI containing	12 (15)	11 (15)	1 (12)	

BMI – body mass index, cART – combination antiretroviral therapy, HBV – hepatitis B virus, HCV – hepatitis C virus, SD – standard deviation.

### Laboratory evaluation

**Figure 1:**
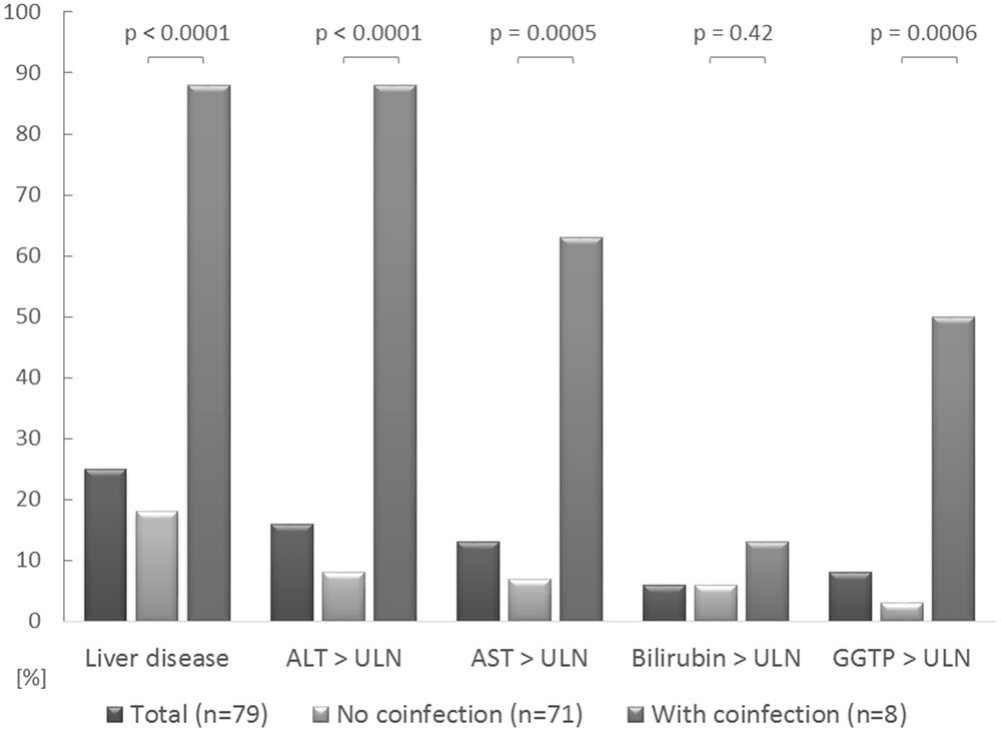
The incidence of the liver marker abnormalities in the studied group and according to the presence of HIV/HBV or HIV/HCV confection. Liver disease was defined as an elevation of any of the following parameters above the upper limit of normal: ALT, AST, bilirubin, GGTP. ALT - alanine aminotransferase, AST - aspartate aminotransferase, GGTP - gamma glutamyl transferase.

Liver disease was diagnosed in 20/79 (25%) of the patients, including 13/71 (18%) of the participants without HBV or HCV coinfection and 7/8 (88%) patients with coinfection (p < 0.0001). Median values of all of the liver markers except bilirubin were significantly higher in the coinfected group compared to patients without coinfection: the median values were 70.5 vs. 25.0 IU/L for ALT (p < 0.0001); 49.0 vs 27.0 IU/L for AST (p = 0.0004), 74.5 vs. 23.0 IU/L for GGTP (p = 0.0002), Table 2. Abnormal ALT, AST, and GGTP values exceeding ULN were significantly more frequently observed in the coinfected group compared to the patients without coinfection (Fig. 1).

**Table 2:** Noninvasive markers of liver disease.

Parameter		Total	HIV without HBV or HCV coinfection Group I	HIV with HBV or HCV coinfection Group II	P Group I vs. Group II
Number		79	71	8	
ALT (IU/L)	Median (IQR)	25.0 (21.3–33.8)	25.0 (21.0–31.0)	70.5 (49.0–83.5)	<0.0001
AST (IU/L)	Median (IQR)	29.0 (24.0–37.0)	27.0 (23.0–35.0)	49.0 (37.0–57.5)	0.0004
Bilirubin (µmol/L)	Median (IQR)	10.6 (7.4–14.9)	10.7 (7.2–15.1)	10.3 (8.9–12.9)	0.94
GGTP (IU/L)	Median (IQR)	24.0 (18.0–38.0)	23.0 (18.7–35.0)	74.5 (41.5–147.5)	0.0002
APRI score	Mean ± SD	0.28 (0.21–0.36)	0.26 (0.20–0.33)	0.57 (0.40–0.75)	0.0001
	>0.5	4 (5)	0	4 (50)	<0.0001
	>1.5	1 (1)	0	1 (13)	0.1
FIB-4	Mean ± SD	0.30 (0.21–0.43)	0.29 (0.19–0.41)	0.50 (0.40–0.60)	0.0025
	>1.45	1 (1)	0	1 (13)	0.1

ALT – alanine aminotransferase, AST – aspartate aminotransferase, APRI – aspartate transferase to platelet ratio index, FIB-4 – Fibrosis-4 index, GGTP – gamma glutamyl transferase, HBV – hepatitis B virus, HCV – hepatitis C virus. Upper limits of normal (ULN) were as follows: 40 IU/L for ALT and AST, 22 µmol/L for bilirubin, and 73 IU/L for GGTP.

### Evaluation of liver fibrosis

Noninvasive evaluation of liver fibrosis based on a calculation of the serum biomarkers showed elevated values of APRI suggesting significant fibrosis (>0.5) in 4/79 (5%) of patients, all of them with HBV or HCV coinfection. In one adolescent with HCV coinfection, the APRI value was 4.84, which indicated cirrhosis. The same patient had an FIB-4 score over 1.45, which suggested significant liver fibrosis (Table 2). The mean values of both APRI and FIB-4 were significantly higher in participants with coinfection than in patients with HIV monoinfection: 0.57 vs. 0.26 (p = 0.0001) and 0.50 vs. 0.29 (p = 0.0025), respectively (Table 2).

### Predictors of the liver disease

Multiple regression analysis revealed that coinfection with either HBV or HCV was independently positively associated with higher values of ALT, AST, GGTP, APRI, and FIB-4. In addition, detectable HIV viral load was positively associated with ALT, AST, bilirubin, APRI, and FIB-4 score values (Table 3). The duration of cART was inversely associated with the ALT level (Table 3).

**Table 3:** Predictors of the liver disease in children with HIV infection (multiple regression analysis).

Predictor	Model for ALT		Model for AST		Model for bilirubin		Model for GGTP		Model for APRI		Model for FIB-4	
	β (SE)	p value	β (SE)	p value	β (SE)	p value	β (SE)	p value	β (SE)	p value	β (SE)	p value
Coinfection with HBV or HCV	73.15 (12.74)	<0.0001	39.00 (7.78)	<0.0001	—	—	88.35 (10.46)	<0.0001	0.64 (0.16)	0.0001	0.20 (0.06)	0.001
Duration of cART	−3,34 (1.44)	0.02	—	—	—	—	—	—	—	—	—	—
Age	3.51 (1.29)	0.008	—	—	−0.34 (0.12)	0.008	—	—	—	—	0.01 (0.004)	0.006
Hemoglobin	—	—	—	—	2.46 (0.47)	<0.0001	4.46 (2.19)	0.04	—	—	—	—
Total cholesterol	—	—	—	—	—	—	0.22 (0.06)	0.0007	—	—	—	—
Platelets	—	—	—	—	—	—	—	—	−0.002 (0.0006)	0.01	−0.001 (0.0003)	<0.0001
HIV viral load (detectable)	58.91 (14.15)	0.0001	41.62 (8.86)	<0.0001	5.47 (2.22)	0.01	—	—	0.75 (0.17)	0.0001	0.21 (0.06)	0.002
BMI z-score	—	—	—	—	−1.29 (0.59)	0.03	—	—	—	—	—	—
**Model performance**												
R^2^	0.47		0.40		0.33		0.55		0.40		0.61	
Adjusted R^2^	0.44		0.38		0.29		0.53		0.38		0.59	

Six separate multivariate models were constructed for the following markers of the liver disease: ALT, AST, bilirubin, GGTP, APRI, and FIB-4. Candidate predictors (age, sex, BMI z-score, CD4 count, duration of cART, haemoglobin level, platelet count, total cholesterol, HBV or HCV coinfection, viral load, and AIDS history) were entered into the model, regardless of the results of the univariate analysis. After entering all variables into the model, the variables that showed least significant associations were subsequently excluded until all variables remained significant (*p* < 0.05). Only predictors showing significance were presented. β – coefficient, SE – standard error. ALT – alanine aminotransferase, AST – aspartate aminotransferase, APRI – aspartate transferase to platelet ratio index, cART – combination antiretroviral therapy, FIB-4 – Fibrosis-4 index, GGTP – gamma glutamyl transferase, HBV – hepatitis B virus, HCV – hepatitis C virus.

The analysis of the influence of different antiretroviral regimens and drugs on liver function parameters in patients without coinfection revealed that regimens containing 2 NRTIs + NNRTI (NVP or EFV) were negatively associated with the level of the bilirubin and positively with the GGTP value, whereas regimens containing PIs (LPV/r or DRV/r) were positive predictors of the bilirubin level and negative for GGTP (Table 4). Analysis of the influence of particular antiretroviral drugs on the liver function parameters is shown in Table 4.

**Table 4:** The influence of different antiretroviral regimens and drugs on liver function parameters in 71 children without HBV or HCV coinfection.

parameter	Number of patients (%)	ALT	AST	bilirubin	GGTP	APRI	FIB-4
Drug/regimen		β (SE), p value	β (SE), p value	β (SE), p value	β (SE), p value	β (SE), p value	β (SE), p value
2 NRTI + NNRTI	32 (45)	—	—	−5.85 (1.26), <0.0001	13.76 (3.34), 0.0001	—	—
2 NRTI + PI	28 (39)	—	—	6.62 (1.24), <0.0001	−9.69 (3.60), 0.009	—	—
3TC	55 (77)	—	5.62 (2.52), 0.02	—	—	—	—
ZDV	24 (34)	—	5.31 (2.21), 0.01	—	—	0.05 (0.02), 0.02	—
TDF	10 (14)	—	−8.35 (2.97), 0.006	—	—	—	—
NVP	18 (25)	—	—	—	15.14 (3.81), 0.0002	—	−0.10 (0.03), 0.007
EFV	14 (20)	—	−7.53 (2.59), 0.004	−6.07 (1.65), 0.0005	—	—	0.11 (0.04), 0.01
LPV/r	28 (39)	—	—	5.91 (1.29), <0.0001	−12.51 (3.46), 0.0006	—	—
DRV/r	5 (7)	—	−8.47 (4.14), 0.04	—	—	—	—

No influence on any of the parameters was found for regimens containing INSTI. β – coefficient, SE – standard error. ALT – alanine aminotransferase, AST – aspartate aminotransferase, APRI – aspartate transferase to platelet ratio index, FIB-4 – Fibrosis-4 index, GGTP – gamma glutamyl transferase, INSTI – integrase inhibitor, NRTI – nucleoside reverse transcriptase inhibitor, NNRTI non-nucleoside reverse transcriptase inhibitor, PI – protease inhibitor.

## Discussion

In this study, we demonstrated that a quarter of HIV-infected children and adolescents at our site have liver test abnormalities. According to other authors, most liver disease among HIV-infected patients is secondary to coinfection with HCV and/or HBV^4,23^. Since these viruses share the same transmission routes, HBV and HCV infections in HIV-infected patients are more common than in general populations^4^. Most children with coinfections acquire them vertically from their mothers^24,25^.

It is estimated that among HIV-infected children, the prevalence rate of chronic hepatitis B may be as high as 49% in some regions, which is significantly higher than the 2–5% in HIV-negative individuals in high prevalence countries^24^. In Poland, the estimated prevalence in the general population is 1–2% for HCV and 2% for HBV. However, almost all children born after 1996 have been vaccinated against hepatitis B in infancy, and the prevalence of HBV infection in children is close to 0. In our cohort of HIV-infected patients, 2% of participants were infected with HBV (all infected vertically from an infected mother). Data on HIV/HBV-coinfected children and adolescents are limited. However, studies on adult patients show that HIV/HBV-coinfected patients have more active liver disease and progress more quickly to liver fibrosis, cirrhosis and end-stage liver disease compared to patients with mono-infection, even though cART is also active against HBV^24^. In addition, HIV/HBV coinfection reduces the rate of spontaneous HBsAg and HBeAg seroconversion, leading to a higher prevalence of HBeAg positive hepatitis B cases and elevated HBV DNA levels, which may increase the risk of progression to cirrhosis and hepatocellular carcinoma^26,27^. Conversely, HBV coinfection does not substantially influence the progression of HIV infection and suppression or the CD4 cell response following cART^24^. Our results suggest that a similar pattern is found in coinfected children and adolescents.

It is estimated that worldwide, approximately 20–30% of all HIV-infected individuals have chronic hepatitis C^28^. Several adult studies have demonstrated that HIV infection modifies the natural history of hepatitis C, leading to an increased probability of chronic infection, higher HCV viral load, and a quicker progression to an end-stage liver disease^29–31^. A meta-analysis of 8 studies in HIV/HCV-coinfected patients revealed a two-fold increased risk for cirrhosis and a five-fold increased risk for decompensated liver disease compared to HCV-monoinfected individuals^32^. Thus, chronic hepatitis C is considered the main cause of liver disease and mortality among HIV-infected adult patients^25,28^. A recent European study on 229 HIV/HCV-coinfected children and adolescents showed that 55% of patients had elevated ALT and 61% had increased AST levels over the ULN, which was higher than the 30% reported for HCV-monoinfected children from the European Paediatric HCV Network^25,33^. The results of our study (showing 88% of children with coinfection presenting with liver test abnormalities) confirm that liver disease is more common in HIV/HCV and HIV/HBV-coinfected children and adolescents than in those with monoinfection. Thus, chronic hepatitis B or C in HIV-infected children may be considered as potentially aggressive infections leading to severe and rapidly progressing liver damage^25^.

Due to the limitations of liver biopsies, alternative non-invasive methods were evaluated to determine liver fibrosis. Recently, several studies on APRI and FIB-4 evaluation in HIV-infected children from the US, Asia and Latin America were published, but similar studies from Europe are lacking^5,6,16,17^. The results of our study are similar to those reported for US children in two different studies (6.5 and 10% with APRI >0.5; 0.8 and 2% with APRI >1.5) and were slightly lower than those reported in a Latin American study (3.2% with APRI >1.5)^6,16,17^. In an Asian cohort, median APRI was 0.34 (0.18–0.63) and was 0.29 (0.05–29.67) in the Latin American study, which is comparable with an APRI 0.28 (0.21–0.36) in our study^5,17^. In Asian children, 2.68% had FIB-4 score >1.3 prior to cART and another 6/820 developed FIB-4 >1.3 during cART follow-up^5^. In a study by Kapogiannis *et al*., among US HIV-infected youth without HBV or HCV coinfection, the authors demonstrated that progression of FIB-4 to more than 1.5 and more than 3.25 was 1.6 (1.2–2.2) and 0.3 (0.2–0.6) cases per 100 person-years, respectively. They also showed that uncontrolled HIV replication was predictive of higher APRI and FIB-4 scores over time^6^. This is in concordance with our results, showing that a detectable viral load was an independent predictor of higher ALT, AST, bilirubin, APRI, and FIB-4 values. This finding is also consistent with the observations by Siberry *et al*., who demonstrated that high viral load, low CD4, and lack of cART were associated with elevated APRI^17^. Our study did not reveal any association between CD4 count and the parameters of liver function; however, the vast majority of children in our cohort had high levels of CD4, which could bias the obtained results.

It has also been shown that longer and better antiretroviral treatment does not increase the risk of elevated APRI, but leads to a lower APRI score^16,17^. In our study, duration of cART was negatively associated with ALT values, which together with the positive association between detectable viral load and liver parameters may suggest that longer and effective cART is protective against liver disease. While the dominant effect of cART seems to be beneficial for liver disease, the potential for toxicity related to specific antiviral drugs should be considered, particularly in younger children, in whom the novel and safer antiviral drugs are unavailable^16^. The incidence of cART-related severe hepatotoxicity is estimated at 10% and liver toxicity is one of the most frequent serious adverse events associated with cART^4,34,35^. Clinical presentation ranges between a mild asymptomatic increase in aminotransferase levels and overt liver failure^36^. All antiretroviral drugs present some risk of hepatotoxicity of a different grade and have characteristic patterns of the liver injury^4^. In a study in Latin American children, the prevalence of elevated APRI varied significantly according to the cART regimen and was estimated to be 3.2% in children on non-PI regimens and 1.5% among children receiving PI-based cART^17^. In our study, the influence on liver function parameters varied according to the particular antiretroviral drug used. In general, PI-based regimens (containing LPV/r) were associated with lower GGTP serum levels and, as expected, higher bilirubin levels, whereas regimens based on NNRTIs (mainly NVP) had an inverse effect on these parameters.

Despite the new data on liver disease in HIV-infected children and adolescents presented in this study, several limitations should be noted. The first issue is its retrospective nature, which did not allow causes and effects to be distinguished. The second issue is a relatively small number of patients in the study group. Liver biopsies were not performed to validate the results of the non-invasive assessment. We did not analyse the results of the ultrasonography. The transient elastography was performed in only two patients, and therefore these results were not included in this study. In addition, we did not analyse the incidence of liver disease progression over time. Thus, this cross-sectional study may under- or over-report the HIV viral load and hepatic endpoints.

In conclusion, HIV-infected children and adolescents with chronic hepatitis B or C are at higher risk for liver disease compared to patients with monoinfection. Effective antiretroviral treatment is beneficial for liver disease, but the potential for toxicity related to specific antiretroviral drugs should be considered. Validation of the non-invasive methods used for evaluation of the liver disease is necessary in children who face a lifetime HIV infection.

## References

[1] Palella FJ (2006). Mortality in the highly active antiretroviral therapy era: changing causes of death and disease in the HIV outpatient study. J Acquir Immune Defic Syndr.

[2] Smith C (2010). Factors associated with specific causes of death amongst HIV-positive individuals in the D:A:D Study. AIDS.

[3] Brady MT (2010). Declines in mortality rates and changes in causes of death in HIV-1-infected children during the HAART era. J Acquir Immune Defic Syndr.

[4] Price JC, Thio CL (2010). Liver disease in the HIV-infected individual. Clin Gastroenterol Hepatol.

[5] Aurpibul L (2015). Prevalence and incidence of liver dysfunction and assessment of biomarkers of liver disease in HIV-infected Asian children. Pediatr Infect Dis J.

[6] Kapogiannis BG (2016). Prevalence of and progression to abnormal noninvasive markers of liver disease (aspartate aminotransferase-to-platelet ratio index and Fibrosis-4) among US HIV-infected youth. AIDS.

[7] Bruno R (2010). gp120 modulates the biology of human hepatic stellate cells: a link between HIV infection and liver fibrogenesis. Gut.

[8] Munshi N, Balasubramanian A, Koziel M, Ganju RK, Groopman JE (2003). Hepatitis C and human immunodeficiency virus envelope proteins cooperatively induce hepatocytic apoptosis via an innocent bystander mechanism. J Infect Dis.

[9] Cappell MS (1991). Hepatobiliary manifestations of the acquired immune deficiency syndrome. Am J Gastroenterol.

[10] Lefkowitch JH (1994). Pathology of AIDS-related liver disease. Dig Dis.

[11] Pol S, Lebray P, Vallet-Pichard A (2004). HIV infection and hepatic enzyme abnormalities: intricacies of the pathogenic mechanisms. Clin Infect Dis.

[12] Crum-Cianflone N (2010). Prevalence and factors associated with liver test abnormalities among human immunodeficiency virus-infected persons. Clin Gastroenterol Hepatol.

[13] Dezsofi, A. *et al*. Liver Biopsy in Children: Position Paper of the ESPGHAN Hepatology Committee. *Journal of pediatric gastroenterology and nutrition*, doi:10.1097/MPG.0000000000000632 (2014). 10.1097/MPG.0000000000000632 25383787

[14] Pokorska-Śpiewak M, Kowalik-Mikołajewska B, Aniszewska M, Pluta M, Marczyńska M (2015). Is liver biopsy still needed in children with chronic viral hepatitis?. World J Gastroenterol.

[15] Castera L (2012). Noninvasive methods to assess liver disease in patients with hepatitis B or C. Gastroenterology.

[16] Siberry GK (2014). Elevated aspartate aminotransferase-to-platelet ratio index in perinatally HIV-infected children in the United States. Pediatr Infect Dis J.

[17] Siberry GK (2014). Prevalence and predictors of elevated aspartate aminotransferase-to-platelet ratio index in Latin American perinatally HIV-infected children. Pediatr Infect Dis J.

[18] WHO recommendations on the diagnosis of HIV infection in infants and children. World Health Organisation, Available at http://apps.who.int/iris/bitstream/10665/44275/1/9789241599085_eng.pdf. Accessed 2017/03/06 (2010). 23741779

[19] Wai CT (2003). A simple noninvasive index can predict both significant fibrosis and cirrhosis in patients with chronic hepatitis C. Hepatology.

[20] Sterling RK (2006). Development of a simple noninvasive index to predict significant fibrosis in patients with HIV/HCV coinfection. Hepatology (Baltimore, Md.).

[21] Lin ZH (2011). Performance of the aspartate aminotransferase-to-platelet ratio index for the staging of hepatitis C-related fibrosis: an updated meta-analysis. Hepatology (Baltimore, Md.).

[22] Centers for Disease Control and Prevention. 1994 Revised classification system for human immunodeficiency virus infection in children less than 13 years of age; Official authorized addenda: human immunodeficiency virus infection codes and official guidelines for coding and reporting ICD-9-CM. MMWR 43(No.RR-12) (1994).

[23] Weber R (2006). Liver-related deaths in persons infected with the human immunodeficiency virus: the D:A:D study. Arch Intern Med.

[24] Healy SA, Gupta S, Melvin AJ (2013). HIV/HBV coinfection in children and antiviral therapy. Expert Rev Anti Infect Ther.

[25] EuroCoord EPHC-ISGITEPAPHCCEI (2017). Coinfection with HIV and hepatitis C virus in 229 children and young adults living in Europe. AIDS.

[26] Colin JF (1999). Influence of human immunodeficiency virus infection on chronic hepatitis B in homosexual men. Hepatology.

[27] Thio CL (2003). Treatment of chronic hepatitis B in the HIV-infected patient. Hopkins HIV Rep.

[28] Soriano V, Barreiro P, Sherman KE (2013). The changing epidemiology of liver disease in HIV patients. AIDS Rev.

[29] Danta M (2008). Impact of HIV on host-virus interactions during early hepatitis C virus infection. J Infect Dis.

[30] Greub G (2000). Clinical progression, survival, and immune recovery during antiretroviral therapy in patients with HIV-1 and hepatitis C virus coinfection: the Swiss HIV Cohort Study. Lancet.

[31] Soto B (1997). Human immunodeficiency virus infection modifies the natural history of chronic parenterally-acquired hepatitis C with an unusually rapid progression to cirrhosis. J Hepatol.

[32] Graham CS (2001). Influence of human immunodeficiency virus infection on the course of hepatitis C virus infection: a meta-analysis. Clin Infect Dis.

[33] European Paediatric Hepatitis, C. V. N. Three broad modalities in the natural history of vertically acquired hepatitis C virus infection. *Clinical infectious diseases: an official publication of the Infectious Diseases Society of America* **41**, 45–51, doi:CID35334 [pii] (2005). 10.1086/430601 15937762

[34] Reisler RB, Han C, Burman WJ, Tedaldi EM, Neaton JD (2003). Grade 4 events are as important as AIDS events in the era of HAART. J Acquir Immune Defic Syndr.

[35] Sulkowski MS, Thomas DL, Chaisson RE, Moore RD (2000). Hepatotoxicity associated with antiretroviral therapy in adults infected with human immunodeficiency virus and the role of hepatitis C or B virus infection. JAMA.

[36] McGovern B (2004). Hepatic safety and HAART. J Int Assoc Physicians AIDS Care (Chic).

